# Convolutional ensembles for Arabic Handwritten Character and Digit Recognition

**DOI:** 10.7717/peerj-cs.167

**Published:** 2018-10-15

**Authors:** Iam Palatnik de Sousa

**Affiliations:** Department of Electrical Engineering, Pontifícia Universidade Católica do Rio de Janeiro, Rio de Janeiro, Brazil

**Keywords:** Offline character recognition, Arabic Handwriting Recognition, Convolutional Neural Networks, Deep learning

## Abstract

A learning algorithm is proposed for the task of Arabic Handwritten Character and Digit recognition. The architecture consists on an ensemble of different Convolutional Neural Networks. The proposed training algorithm uses a combination of adaptive gradient descent on the first epochs and regular stochastic gradient descent in the last epochs, to facilitate convergence. Different validation strategies are tested, namely Monte Carlo Cross-Validation and K-fold Cross Validation. Hyper-parameter tuning was done by using the MADbase digits dataset. State of the art validation and testing classification accuracies were achieved, with average values of 99.74% and 99.47% respectively. The same algorithm was then trained and tested with the AHCD character dataset, also yielding state of the art validation and testing classification accuracies: 98.60% and 98.42% respectively.

## Introduction

Offline handwriting recognition refers to the task of determining what letters or digits are present in a digital image of handwritten text. It is considered a subtask of the more general Optical Character Recognition. However, in many applications, from reading bank checks to postal mail triage, offline recognition plays a key role, greatly motivating the development of accurate and fast classification algorithms (Abdelazeem, 2009).

The domain of Arabic Handwriting Recognition (AHR), however, has only been explored in depth more recently. Younis (2017) notes that AHR suffers from slow development compared to Handwriting Recognition in other languages. He further mentions that Arabic characters contain a specific set of challenges that make the task more difficult. Such difficulties include the positioning of dots relative to the main character, the variability caused by the use of the characters in multiple countries and different areas of knowledge and work, among others.

Given this issue, using datasets that represent this variability on a large number of images is essential.

In the previous decade, a dataset equivalent to MNIST (LeCun et al., 1998) was developed to allow for a more direct comparison of the performance of classification algorithms on Latin and Arabic digits.

This dataset was named MADbase (Abdleazeem & El-Sherif, 2008), and consists of 70,000 images of Arabic digits, written by 700 participants from different areas of work and backgrounds. These are divided into a training set of 60,000 images and a test set of 10,000. This seems to be the largest dataset for this task available in literature. This makes it an ideal choice for training the network and fine-tuning parameters. Furthermore, as discussed in detail on the next section, previous results obtained from this dataset allow for comparison with the results presented in this manuscript. It is worth noting that this dataset is a modified version of an equivalent dataset called ADbase, which contains the same images with a different image size. To create MADbase, ADbase images were resized and transformed from binary to grayscale to be equivalent to MNIST.

While the MADbase dataset deals with digits, the Arabic Handwritten Character Dataset (AHCD) (El-Sawy, Loey & Hazem, 2017) includes 16,800 images of isolated characters divided in training set of 13,440 and a test set of 3,360 images. This seems to be the largest dataset available for this classification task.

Regarding previous results, Mahmoud (2008) presented a method for recognition of handwritten Arabic digits based on extraction of Gabor-based features and Support Vector Machines (SVMs). The dataset used in this case contained 21,120 samples provided by 44 writers. The average classification accuracy rates obtained were of 99.85% and 97.94% using three scales & five orientations and four scales & six orientations respectively.

Abdleazeem & El-Sherif (2008) applied several classification methods to the MADbase dataset. Their best result was obtained with a Radial Basis Function Support Vector Machine (RBF SVM), with which a two stage classification was performed. In the first stage several customized features were extracted from a similar dataset by the researchers, and then used as input for the RBF SVM. The classifier was tuned to maximize the classification accuracy, which had a final value of 99.48%. This value corresponds to the best parameter combination.

El Melhaoui et al. (2011) used a small dataset of 600 digit images to obtain a 99% recognition rate using a technique based Loci characteristics.

Pandi Selvi & Meyyappan (2013) proposed an approach for Arabic Digit recognition using neural networks and training through backpropagation. The dataset used in this case was also small, and the classification accuracy obtained was 96%.

Takruri, Al-Hmouz & Al-Hmouz (2014) obtained a test classification accuracy of 88% using a dataset of 3,510 digit images, by using a three level classifier consisting on SVM, Fuzzy C Means and Unique Pixels.

Salameh (2014) presented two methods for enhancing recognition of Arabic Handwritten Digits. The methods combine fuzzy logic pattern classification to counting the number of ends of the digit shapes to obtain a classification test accuracy of 95% for some fonts.

Alkhateeb & Alseid (2014), using the ADbase dataset, obtained an 85.26% classification accuracy by using Dynamic Bayesian Networks (DBN).

Although it is hard to compare results provided by training with different datasets, the larger datasets seem to result in worse classification accuracies, most likely since they cover a larger sample of the variability of styles in handwriting. This further indicates that using the largest, more challenging datasets available, with the largest number of writing participants, is an ideal choice, as was done for this manuscript.

Loey, El-Sawy & EL-Bakry (2017) used Stacked Autoencoders on the MADbase dataset to obtain a classification accuracy of 98.5%.

Mudhsh & Almodfer (2017) obtained a validation accuracy of up to 99.66% on the MADbase dataset by usage of dropout regularization and data augmentation, and an architecture inspired by the VGGNet Convolutional Neural Network (CNN) (Simonyan & Zisserman, 2014). Importantly, they mention in the text that this validation accuracy does not hold for the test set, without mentioning explicitly the test accuracy. The validation method was a 10-fold cross-validation. They also tested the performance of the algorithm on a dataset of 6,600 images of characters, obtaining a validation accuracy of 97.32%. Again they mention that this validation accuracy does not hold for the test set, without clearly stating the test accuracy.

Younis (2017) obtained an accuracy of 97.60% on the previously mentioned AHCD dataset, by use of a Deep CNN with batch normalization and learning rate scheduling.

The general trend observed in these works is that feature extraction aids in the classification task. This makes the choice of convolution based networks straightforward, as these architectures are precisely constructed to be specialized feature extractors. It seems to make sense that CNNs have the best results so far for this task, in these previously reported results.

In this work, the best previous ideas and results are incremented further by usage of some changes in architecture and in the training procedure. Namely both the VGGNet inspiration and a batch normalized CNN are employed, combining their classifications through ensemble averaging. The details of this method are described in the next section.

## Materials and Methods

The code for defining and training the networks was implemented in Python, using the Keras framework with Tensorflow backend. The key aspects of the classification system are, namely, the selection and preparation of the datasets, the network architecture, the training schedule, the validation strategy, the data augmentation, and the ensemble selection. Each of these is explained more in detail below.

### Selection and preparation of datasets

The datasets chosen for training and parameter tuning were the MADbase and AHCD datasets described in the previous section. For both datasets, the networks were trained from scratch, although most of the parameter tuning was done with MADbase, since it is a much larger dataset.

**Figure 1:**
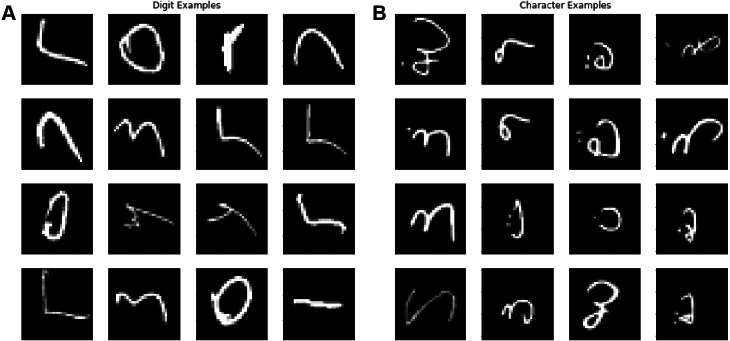
Example images from the MADbase and AHCD datasets.

Since the images in both datasets are already prepared for training, the only pre-processing done was converting the value of each pixel to float format and dividing by 255 for normalization purposes. Figure 1 shows some examples from each dataset.

### Network architecture

The classification method consists on an ensemble of four networks. These are actually two networks, each trained with two different strategies (with data augmentation and without). Rather than choosing whether to apply augmentation or not, both options are used and the results are gathered in an ensemble classifier. This also allows for a direct comparison of the predictive power of each individual network against the results of the ensemble. For brevity, the ensemble of four networks will be called *ENS4* throughout the manuscript.

The first type of CNN used in the ensemble was inspired by the VGG16 network, readily implemented for Keras. This architecture couldn’t be used directly, however, because it assumes the inputs are images of three channels (RGB) of default size 224 by 244 pixels, and minimum size 48 by 48 pixels. Images below this size are too small to pass through the five convolution blocks of the Network. The images of MADbase and AHCD have dimensions of 28 by 28 pixels and 32 by 32 pixels, respectively. Furthermore they are grayscale images with only 1 channel.

The solution to this was adapting the VGG16 architecture by removing the fifth convolution block, and creating three channel images from the one channel images by simply stacking the same single channel three times. Another adaptation added was a dropout layer before the final dense softmax layer, and only using two dense layers instead of three. The resulting 12 layer architecture, intended for these grayscale images, will be called *VGG12* on this manuscript, for brevity.

The second type of CNN used was designed from scratch in this work to include the dropout and batch normalization regularizations within both the feature extraction convolution blocks as well as the dense fully connected classification block. The architecture was adapted after several experiments to be as simple as possible, allowing for fast training, while still providing robust classifications. For brevity this architecture that includes both types of regularizations (dropout and batch normalization) will be termed *REGU* throughout the rest of the manuscript.

**Figure 2:**
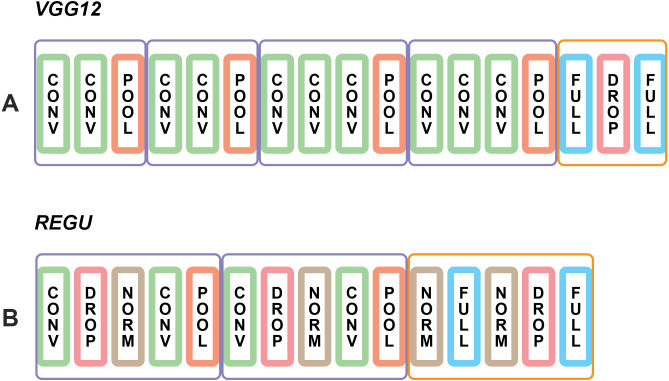
Diagrams of *VGG12* and *REGU*. CONV refers to convolutional layers, POOL to max-pooling layers, FULL to dense fully connected layers and NORM to batch normalization layers. Purple round rectangles correspond to convolutional feature extraction blocks, and orange round rectangles correspond to fully connected classification blocks. More details about the architectures can be found on a suplemental file along the manuscript.

Figure 2 contains illustrative schemes of *VGG12* and *REGU*.

Namely, *VGG12* contains four convolution blocks and one fully connected block. The convolution filters used in all convolution layers have size 3 × 3. The number of filters used in the first block is 64, and doubles on every further block up to 512 on block 4. *ReLU* activation was used for the convolution layers, as well as same padding. The max pooling elements had a size of 2 × 2.

The fully connected block has two dense layers. The first, with *ReLU* activation, has 512 neurons. The second, with *softmax* activation, has 10 neurons for the case of MADbase and 28 for the case of AHCD. A 0.25 dropout rate was used.

Regarding *REGU*, there are two convolution blocks and one fully connected block. The first convolution block has two convolution layers with 32 filters of size 3 × 3 and *ReLU* activation. A 0.2 dropout rate was used, followed by Batch Normalization. The max pooling elements had a size of 2 × 2. The second convolution block is identical, except for the number of convolution filters, which is 64 and for a Batch Normalization applied at the start of the block.

The fully connected block in this case has Batch Normalizations before each dense layer. The dense layers are identical to the case of *VGG12*. The first has 512 neurons and *ReLU* activation, and the second has *softmax* activation and 10 neurons for MADBase and 28 neurons for AHCD. A 0.2 dropout rate was used. These descriptions are summarized in a supplemental file that can be used for building this architecture on Keras.

### Training schedule

The literature review and previous works cited show that, in general, the training for AHR tasks is done with optimizers such as Stochastic Gradient Descent (SGD) or with an adaptive method such as Adam (Kingma & Ba, 2014), often paired with learning rate schedules.

However, a generalization gap between Adam and SGD has been observed recently for many tasks involving image classification and language modelling (Keskar & Socher, 2017). It has been indicated that that SGD finds more optimal lower minimums for the loss function, despite converging at a much lower rate than Adam. According to Keskar and Socher, this favors the usage of Adam for the first epochs of training, providing a fast convergence to lower losses, with a swap to SGD for a more fine convergence at the end of the training. This swapping strategy closed the generalization gap on the tests performed by Keskar and Socher.

A few experiments were performed with *VGG12* and *REGU* using Adam, SGD and this Adam and SGD swapping strategy. The initial results confirmed the observations of Keskar and Socher, and as such the swapping strategy was adopted for the rest of the experiments.

Namely, the number of epochs before and after the swap was treated as a parameter to be tuned, and eventually values of 20 epochs of Adam training followed by 20 epochs of SGD training seemed to provide the best results.

For the 20 epochs of SGD training, inspired by previous works that used learning rate scheduling, a strategy of reducing the learning rate periodically was adopted. Specifically, this only happened if and when the test loss reached a plateau. There is an already implemented function in Keras, *ReduceonLRPPlateau*, for this purpose. Whenever a plateau was reached, the learning rate was multiplied by a factor of 0.1.

For this task in particular, the choice of this training strategy produced better results when compared to use of SGD or Adam individually for 40 epochs. It is the first time such a strategy has been employed for the task of Arabic Handwritten Character and Digit Recognition.

It is also worth noting that using SGD individually didn’t reliably give similar results to the swapping strategy, even when more training epochs were allowed, as SGD seemed to have trouble converging on the first few epochs of training, remaining at high training and validation and loss values.

The loss function used was Categorical Cross-entropy, which is adequate given the softmax activation of the last dense layer of both *VGG12* and *REGU*. Mean square error was also tried in initial experiments, but it consistently resulted in worse performance.

### Validation strategy

Both datasets used (MADbase and AHCD) provide separate test sets, but not separate validation sets. If the test sets were to be used for validation purposes, this would make the classifier heavily biased towards that specific test set. It would then be difficult to verify how good the classifier is at generalizing.

As such, using part of the training set for validation is the ideal approach. With the validation set all the parameters were tuned to find the highest values of validation accuracy, and only after this training was done, and no other changes were to be effected to *ENS4*, the testing set was used for evaluation.

However this means that the validation strategy chosen for parameter tuning could affect the generalization capabilities of the network. Furthermore, there is randomness present in the training procedure, whether in weight initialization, in the data augmentation method, the dropout regularization or other aspects. This suggests that multiple runs are necessary to obtain an average behavior and performance of the classifier.

The most commonly applied validation methodologies that use multiple runs are Monte Carlo Cross-Validation (MCCV) (Xu & Liang, 2001) and K-fold Cross-Validation (KCV) (Refaeilzadeh, Tang & Liu, 2016).

In MCCV, a subset of the training set is chosen at random and used as a validation set. This is repeated as many times as necessary, in general ensuring that the validation set always has the same size.

In KCV the training set is divided into *K* subsets (named folds) of the same size, and each fold is used as a validation set, while all of the other folds are gathered as a training set. A very commonly used value for *K* is 10.

Generally speaking there isn’t a definitive answer as to which of these two methodologies is best for a given task, as this is highly dependent on the particularities of each dataset. Mudhsh & Almodfer (2017), for instance, have used 10-fold cross validation in their study of MADbase.

For this present manuscript, both MCCV and KCV were employed for the MADbase dataset to give as much information as possible for fine-tuning the parameters, before the test set was used for evaluation. Since the test set has 10,000 images for MADbase, the MCCV was implemented so that the validation sets also had 10,000 images. This means the training sets effectively had 50,000 images during training. A total of 10 runs were performed in this manner, and the average performances were computed.

For the KCV, a 10-fold Cross-Validation was used to allow for direct comparison with the results of Mudhsh & Almodfer (2017), but it must be noted that dividing the original training set of 60,000 into 10 folds means each validation set has a size of 6,000. Since the size of the validation set can be adjusted by changing the value of *K*, and the test set size is fixed, a 6-fold validation was also performed (since this implies validation sets of size 10,000, the same as the provided test sets).

Given the smaller size of AHCD, using 10-fold cross validation makes the validation sets too small compared to the test set, and as such only MCCV was employed in that case, ensuring the validation and test sets had the same size. As with MADbase, this cross-validation was repeated 10 times.

The results of the several runs with each method were averaged to allow for decision making regarding parameter tuning. Once the best validation results were reached with *ENS4*, the test set was used for evaluation.

### Data augmentation

The method of data augmentation has been used previously in AHR (Mudhsh & Almodfer, 2017). In the present study, data augmentation was applied to the training sets of both MADbase and AHCD in some of the experiments. The purpose is to create a more varied dataset that could make the classifier more robust. Since *ENS4* includes both the networks trained without and with data augmentation, the networks corresponding to the latter case will be named *VGG12_aug* and *REGU_aug* for disambiguation.

The augmentation method used was the already implemented *ImageDataGenerator* on Keras, with zoom_range, height_shift_range and width shift range parameters equal to 0.1. Other parameters were also tested, but invariably led to a worse performance of the augmented classifiers. It is known that not all forms of augmentation are necessarily helpful for all tasks (Mudhsh & Almodfer, 2017), and the tree chosen yield the best results for these AHR architectures. The batch size of augmented images had a size of 128.

### Ensemble selection

Once *VGG12*, *VGG12_aug*, *REGU* and *REGU_aug* were trained, the idea was to combine their predictions into an averaged ensemble classifier (Simonyan & Zisserman, 2014). The two main approaches that could be used for this include averaging the predictions of each of the 4 networks that form *ENS4*, or using a maximum voting approach, where the biggest softmax probability between the four networks is taken as the answer. Both methods were initially used, with averaging eventually showing a better performance overall.

As such, the output softmax probabilities of *ENS4* are the average calculated from the outputs of *VGG12*, *VGG12_aug*, *REGU* and *REGU_aug.*

Roughly, the process of training the entire ensemble, took about 2 h per run on the available hardware. GPU acceleration was used.

### Weight initialization

Previous works in literature regarding AHR often don’t describe weight initialization strategies. For this study we have used Glorot-Normal initialization (Glorot & Bengio, 2010). On their work, Glorot and Bengio mention how this initialization often outperforms other normalized initializations. Indeed, this came to be the standard initialization for the Keras framework.

For comparison, runs with He-Normal, Random normalized and All-zeroes initializations were performed. Preliminary tests showed that the standard Glorot-Normal initialization yielded better results, and so this was kept throughout the rest of the runs.

## Results

**Figure 3:**
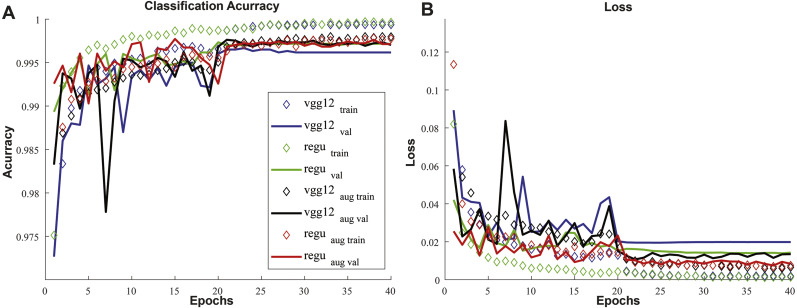
Example of training and validation acurracy and loss as a function of training epochs. Each of the four individual networks that compose ENS4 are portrayed. Up to the 20th epoch, under Adam optimization, the values gradually oscilate less. Notably when swapping to SGD at epoch 20 there is a slight improvement of performance (higher acurracy, lower loss), followed by a narrower convergence of the values. This behavior was consistent throughout all runs.

The optimizer swapping strategy described in the previous section, combined with the learning rate scheduling, produces a consistent behavior of convergence of the loss function with the training epochs. In the first twenty epochs, the Adam optimizer causes loss values to drop towards lower and more stable values, and on the next 20 epochs SGD brings these values to a lower, nearly constant minimum. An example of this behavior can be seen in Fig. 3, showing the plots for loss and accuracy over the training epochs.

After the initial parameter tuning was performed with MADbase, the 26 experiments corresponding to 10 MCCV runs, 10 fold runs and six fold runs were performed. The averaged results are summarized in Table 1. The full raw results of the runs, used to calculate these averages, are presented as a supplemental file along the manuscript.

**Table 1:** Summary of results. Averaged test and validation accuracies with different cross- validation strategies. Entries in the table correspond to the individual networks (*VGG12, REGU, VGG12_aug* and *REGU_aug*) and *ENS4*.

	MCCV		KCV 10fold		KCV 6fold	
	Accuracy (%)	Standard Deviation (*n*%)	Accuracy (%)	Standard Deviation (*n*%)	Accuracy (%)	Standard Deviation (*n*%)
****	**Validation (MADbase)**					
*VGG12*	99,56	0,05	99,60	0,06	99,62	0,05
*REGU*	99,61	0,05	99,58	0,06	99,58	0,08
*VGG12_aug*	99,63	0,05	99,59	0,06	99,62	0,06
*REGU_aug*	99,71	0,07	99,69	0,07	99,72	0,07
*ENS4*	99,74	0,06	99,73	0,05	99,74	0,07
	**Test (MADbase)**					
*VGG12*	99,20	0,05	99,23	0,08	99,26	0,07
*REGU*	99,17	0,04	99,15	0,10	99,18	0,05
*VGG12_aug*	99,32	0,04	99,31	0,03	99,32	0,03
*REGU_aug*	99,37	0,04	99,40	0,06	99,39	0,05
*ENS4*	99,43	0,03	99,44	0,04	99,47	0,04

Interestingly, the only case where one of the individual networks outperformed the full ensemble was for one of the *REGU_aug* results. Furthermore, *REGU_aug* consistently outperformed *VGG12_aug* in all experiments with this dataset, even though the architecture is arguably much simpler (having effectively six layers compared to the 12 of *VGG12*).

For MADbase, the maximum value of test accuracy was observed during one of the 10-fold tests: 99.52%. This result outperforms the 99.48% RBF SVM result reported Abdleazeem & El-Sherif (2008). The maximum validation accuracy was observed during one the MCCV runs: 99.86%, which outperforms the 99.66% validation accuracy reported by Mudhsh & Almodfer (2017).

It was also observed that the final averaged test accuracy of 6-fold validation for MADbase was the best result among the three validation strategies. However it surpasses the other two by only 0.02%. In the MADbase test dataset of 10,000 images this corresponds to a difference of just two images. The difference in stdev is also small, of 0.01%. Overall this does not seem to show a clear best choice between MCCV and KCV validation strategies.

As such, the AHCD dataset was studied using MCCV for parameter tuning. The validation and test accuracies were, respectively, 98.60% and 98.42%. These also meet and improve upon the state of the art values mentioned in ‘Introduction’.

## Discussion

Notably, standard deviation (stdev) of the results of 10 MCCV runs were lower than the standard deviation of either KCV. The only exceptions are for the *ENS4* validation stdev, and the *VGG12_aug* test stdev. This seems to indicate that the MCCV yields less disperse validation and test accuracies for MADbase.

The fact that *REGU* was observed to outperform *VGG12* suggests the importance of batch normalization for this task.

It was also observed that data augmentation resulted consistently in improvements for both validation and test accuracies. Furthermore, ensemble averaging resulted in higher validation and test accuracies while at the same time reducing the standard deviation over the number of the experiments performed.

In terms of the validation and test accuracies, 10-fold cross-validation was consistently a worse performing metric compared to sixfold cross-validation and MCCV. Generally speaking whenever tenfold cross-validation was used for parameter tuning, the observed accuracies were in general worse. This is true for both validation and test accuracies.

However for the most part, the observed differences were on the order of less than 10 misclassifications, which doesn’t justify preference for a particular validation strategy if it would be much more computationally costly than the alternative.

The average test and validation accuracy values of *ENS4* are very promising and improve upon the presently available state of the art listed in ‘Introduction’, for MADbase. The best test accuracy result of 99.52% indicates that *ENS4* is the first classifier to outperform the accuracy value of 99.48% of the two stage RBF SVM classifier by Abdleazeem & El-Sherif (2008) for this dataset. Importantly, *ENS4* achieves this in a single stage classification, with no previous feature extraction.

## Conclusion

A method for Offline Arabic Handwritten Recognition was described in this manuscript. The system was trained and tested on the two largest available datasets of Arabic digits and characters. The architecture used consisted of an ensemble averaging of four Convolutional Neural Networks. Of these four, two were inspired by *VGGNet* and two were written from scratch using batch normalization and dropout regularization. Each of these was trained twice: once with data augmentation, once without.

The training used a swapping method where the first epochs use an adaptive optimizer (Adam) and the last epochs use regular stochastic gradient descent. It further used learning rate scheduling if the loss decrease reached a plateau during the SGD training epochs.

Two validation strategies were considered: Monte Carlo Cross-Validation and K-fold Cross-validation. For the latter, two values of *K* were used, one commonly used in literature, and one that ensures the test and validation sets have the same size for the MADbase dataset. The results didn’t show a clear advantage of choosing either method for this dataset in particular.

The use of a categorical cross-entropy loss function outperformed the use of a mean squared error function for the same purpose, possibly because of the choice of softmax activations for the final dense layer of the individual networks.

Glorot-Normal weight initialization outperformed the other alternatives tested (He-Normal, All-zero, Random normalized). Future works could test initializations more exhaustively, to see if there is a particular combination of initializations that yield better results for AHR, although the results so far seem to indicate that other aspects of the architecture and training are more relevant to the end result.

The results obtained improve upon the state of the art both the MADbase and AHCD datasets. The fact the ensemble averaging gives promising results suggests future projects could adapt other types of larger Convolution based Networks, or try different training strategies, while also adding them to ensemble averaging classifiers. Other types of ensemble averaging, such as weighted averages, could be explored more in depth for this purpose as well.

##  Supplemental Information

10.7717/peerj-cs.167/supp-1 Supplemental Information 1 Architecture details This file contains specific details about *VGG12* and *REGU*, such as the shape of filters, number of filters used for convolution, stride used for pooling, type of activation used for each layer, among others.

10.7717/peerj-cs.167/supp-2 Supplemental Information 2 Raw data from runs This file contains the raw acurracy results (validation and testing) for all 26 runs computed: 10 using MCCV, 10 using 10-fold validation, 6 using 6-fold validation.

10.7717/peerj-cs.167/supp-3 Supplemental Information 3 Run script This file contains the template for running the training script. This assumes the datasets have been downloaded already. This particular file runs the 4th fold in the 10-fold Cross-Validation. All other runs were performed similarly by just changing the validation sets manually to the desired fold or equivalent.

10.7717/peerj-cs.167/supp-4 Supplemental Information 4 Adam vs SGD vs SWATS plots 8 plots from premilinary tests of training schedules using only Adam, only SGD, and SWATS (swapping from Adam to SGD). Images cover all networks (REGU, VGG12, REGU_aug, VGG12_aug), in both training and validation. Green Represents SGD-Only training. Red represents Adam-Only training. Black represents SWATS training (the swapping strategy).

## Additional Information and Declarations

### Data Availability

The following information was supplied regarding data availability: There are three supplemental files. The text file includes relevant specific details about the network architectures used in the manuscript. The Excel file includes the raw results from the experiments run in the manuscript. The ipynb file is the Pthon code notebook. The raw data (specific details about the network architectures used, raw results from the experiments run and the Python code notebook) is available as Supplemental Files. AHCD: https://www.kaggle.com/mloey1/ahcd1. MADbase: https://www.kaggle.com/mloey1/ahdd1/.
